# Gradenigo Syndrome: A Case Report of a Rare Complication of Otitis Media

**DOI:** 10.7759/cureus.51865

**Published:** 2024-01-08

**Authors:** Zainab Ali, Sajdah Alhantoosh, Zaynab Albaqal, Rawan Alkhudaimi, Ahlam S Alharbi, Tareq A Hamad

**Affiliations:** 1 General Practice, Imam Abdulrahman Bin Faisal University, Dammam, SAU; 2 Emergency Medicine, Al-Adwani General Hospital, Taif, SAU; 3 Otolaryngology, Al-Adwani General Hospital, Taif, SAU

**Keywords:** unilateral abducens nerve palsy, magnetic resonance imaging, meckel's cave, gradenigo syndrome, otitis media

## Abstract

Otitis media, a common inflammation of the middle ear, frequently complicates upper respiratory infections. Gradenigo's syndrome, a rare complication, manifests as suppurative otitis media, abducens nerve palsy, and severe trigeminal nerve pain. Prompt recognition is vital because of the proximity of the infection to critical neurovascular structures. We present the case of a 38-year-old female who presented with facial pain, otalgia, and diplopia following an upper respiratory infection. Examination revealed facial weakness and abducens nerve palsy. Laboratory results showed elevated inflammatory markers. Imaging confirmed middle ear involvement. Antibiotics were initiated, and myringotomy was performed, resulting in a successful outcome. This case report contributes to understanding Gradenigo's syndrome's clinical nuances, emphasizing the necessity of a structured diagnostic and therapeutic approach. Ongoing research is crucial for refining diagnostic criteria, optimizing treatment, and enhancing pathophysiological understanding. Increased medical education is imperative to ensure early detection and improved patient outcomes.

## Introduction

Otitis media, inflammation of the middle ear, is a common condition, particularly prevalent in the pediatric population. It often arises in the context of upper respiratory infections and can lead to complications when left untreated. Among the potential sequelae of otitis media, Gradenigo's syndrome stands out as a rare and distinctive complication [1]. Gradenigo's syndrome is characterized by the triad of suppurative otitis media, abducens nerve palsy, and severe pain localized to the distribution of the trigeminal nerve. The condition primarily arises because of the extension of infection from the middle ear to the petrous apex of the temporal bone. The anatomical proximity of the petrous apex to crucial neurovascular structures contributes to the unique clinical features of Gradenigo's syndrome, making its early recognition and intervention imperative for optimal patient outcomes [1,2].

The syndrome's name pays homage to Giuseppe Gradenigo, an Italian otologist who first described this clinical entity in the early 20th century [1,2]. While Gradenigo's syndrome is a rare manifestation, understanding its pathophysiology and clinical presentation is crucial for healthcare providers, as delayed diagnosis and management may lead to severe complications, including intracranial involvement.

## Case presentation

A 38-year-old female presented to the emergency department with a three-day history of right-sided facial pain, otalgia, and diplopia. The patient reported an antecedent upper respiratory tract infection two weeks before the onset of her symptoms. She denied any recent trauma, dental procedures, or previous episodes of similar symptoms. The patient had a medical history of chronic sinusitis and recurrent otitis media but had not experienced any recent ear infections.

On physical examination, the patient appeared fatigued and displayed signs of right-sided facial weakness with the inability to close her right eye completely. Otoscopic examination revealed erythema and edema of the right external auditory canal. A thorough neurological examination demonstrated right abducens nerve palsy, contributing to her diplopia. No other cranial nerve abnormalities were noted, and the remainder of the neurological examination was unremarkable.

Given the severity of symptoms and suspicion of an inflammatory process, laboratory investigations were performed. The complete blood count showed a mild leukocytosis with a white blood cell count of 12,500/μL (reference range: 4,000-11,000/μL). Inflammatory markers such as C-reactive protein were elevated at 30 mg/L (reference range: <5 mg/L), and the erythrocyte sedimentation rate was increased to 45 mm/h (reference range: 0-20 mm/h). Cerebrospinal fluid analysis was performed, and the results were unremarkable, ruling out central nervous system infection. Blood cultures were obtained, and broad-spectrum antibiotics were initiated immediately upon admission (Table 1).

**Table 1:** Comprehensive laboratory results of the patient on admission. Indicators: *N* (Normal), *H* (High), and *L* (Low) represent the respective ranges for the laboratory results.

Lab Parameter	Result	Reference Range	Indicator
White Blood Cell Count	12,500/μL	4,000-11,000/μL	H
Hemoglobin	13 g/dL	12-16 g/dL	N
Platelet Count	300,000/μL	150,000-400,000/μL	N
C-Reactive Protein	30 mg/L	<5 mg/L	H
Erythrocyte Sedimentation Rate	45 mm/h	0-20 mm/h	H
Blood Glucose	100 mg/dL	70-100 mg/dL	N
Blood Urea Nitrogen	15 mg/dL	8-20 mg/dL	N
Serum Creatinine	0.8 mg/dL	0.6-1.2 mg/dL	N
Sodium	140 mmol/L	135-145 mmol/L	N
Potassium	4.0 mmol/L	3.5-5.0 mmol/L	N
Total Bilirubin	0.7 mg/dL	0.2-1.2 mg/dL	N
Alanine Aminotransferase	25 U/L	7-56 U/L	N
Aspartate Aminotransferase	30 U/L	10-40 U/L	N
Alkaline Phosphatase	70 U/L	40-129 U/L	N

**Figure 1:**
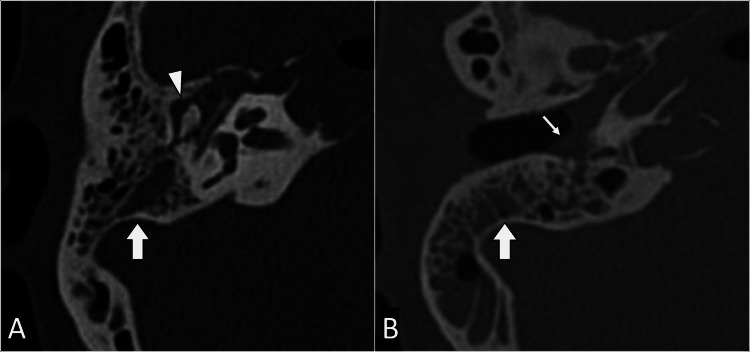
Axial CT images of the right internal acoustic meatus (IAM) in bone (A) and soft tissue (B) windows reveal soft tissue material within the external auditory canal (arrow), accompanied by complete opacification of the mastoid air cells (block arrow) and the presence of middle ear effusion (arrowhead). CT: Computed tomography

**Figure 2:**
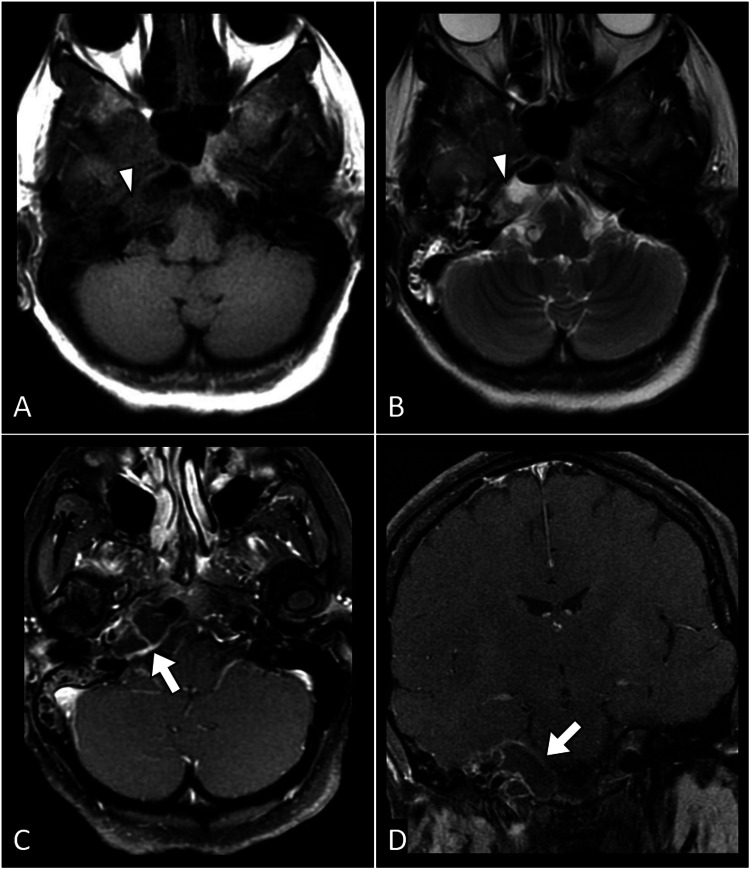
Multiple MR images depicting various sequences. A (axial T1-weighted image), B (axial T2-weighted image), C (axial T1-FS post-contrast), and D (coronal T1-FS post-contrast) illustrate fluid signal intensity within the right petrous apex (arrowhead in A and B), demonstrating peripheral enhancement in the post-contrast image (arrow in C). Additionally, there is the presence of the dural enhancement (arrow in D) involving the right Meckel's cave. MR: magnetic resonance, FS: fat saturation

Further investigations were initiated to confirm the suspected diagnosis. Computed tomography of the internal acoustic meatus revealed fluid accumulation within the right middle ear and mastoid air cells. No bone erosion was noted in the mastoid region, and the ossicles appeared intact without significant involvement (Figure 1). Subsequently, magnetic resonance imaging of the brain with contrast revealed peripheral enhancement around the hypointense fluid, suggesting the presence of a collection. Additionally, thickening and enhancement of the dura around the entrance to Meckel's cave were noted, contributing to the diagnostic evaluation of Gradenigo syndrome (Figure 2).

The patient was started on a regimen of intravenous ceftriaxone (2 g every 12 hours) and metronidazole (500 mg every eight hours) to target potential bacterial pathogens involved in petrous apicitis and mastoiditis. In consultation with the otolaryngology team, a myringotomy was performed to drain the purulent material from the middle ear. The patient was monitored closely for any signs of neurological deterioration.

## Discussion

Gradenigo's syndrome, a rare complication of otitis media, represents a unique clinical entity characterized by the triad of suppurative otitis media, abducens nerve palsy, and severe pain localized to the distribution of the trigeminal nerve. The case of our 38-year-old female patient sheds light on the clinical intricacies and challenges associated with this syndrome, emphasizing the importance of timely recognition and intervention. The rarity of Gradenigo's syndrome, coupled with its potential for serious complications, underscores the significance of heightened clinical awareness among healthcare professionals [1-3].

The clinical presentation of Gradenigo's syndrome often follows an antecedent upper respiratory tract infection, as observed in our patient. The syndrome's pathogenesis involves the extension of infection from the middle ear to the petrous apex of the temporal bone, leading to inflammation and involvement of the adjacent structures. Notably, the proximity of the petrous apex to critical neurovascular structures, such as the abducens nerve and Meckel's cave, contributes to the distinctive clinical features, including facial pain, otalgia, and abducens nerve palsy [3,4].

Diagnostic challenges in Gradenigo's syndrome necessitate a comprehensive approach. In our case, imaging studies played a pivotal role in confirming the diagnosis. Computed tomography revealed fluid accumulation within the middle ear and mastoid air cells, while magnetic resonance imaging with contrast highlighted peripheral enhancement around the fluid collection and dural enhancement around Meckel's cave [2-4]. These findings are consistent with the existing literature, emphasizing the utility of imaging in guiding diagnosis and facilitating timely intervention.

Management of Gradenigo's syndrome involves a multidisciplinary approach. Our patient received prompt intravenous antibiotics targeting potential bacterial pathogens involved in petrous apicitis and mastoiditis. Collaboration with the otolaryngology team led to a myringotomy to drain purulent material from the middle ear [2,5]. The successful outcome in this case reinforces the effectiveness of early and coordinated medical and surgical interventions.

## Conclusions

While Gradenigo's syndrome remains a rare entity, this case report contributes to the growing body of literature highlighting its varied clinical presentations and the importance of a structured diagnostic and therapeutic approach. Further research is warranted to refine diagnostic criteria, explore optimal treatment strategies, and enhance our understanding of the underlying pathophysiology. The rarity of Gradenigo's syndrome underscores the need for continued medical education to ensure that healthcare providers maintain a high index of suspicion, facilitating early diagnosis and improving patient outcomes.
